# Effects of gas sorption-induced swelling/shrinkage on the cleat compressibility of coal under different bedding directions

**DOI:** 10.1038/s41598-017-14678-1

**Published:** 2017-10-30

**Authors:** Shoujian Peng, Zhiming Fang, Jian Shen, Jiang Xu, Geoff Wang

**Affiliations:** 1 State Key Laboratory of Coal Mine Disaster Dynamics and Control, Chongqing University, Chongqing, 400044 China; 2 School of Chemical Engineering, The University of Queensland, Brisbane, QLD 4072 Australia; 3 State Key Laboratory of Geomechanics and Geotechnical Engineering, Institute of Rock and Soil, Mechanics, Chinese Academy of Sciences, Wuhan, Hubei 430071 China; 4 Key Laboratory of CBM Resources and Dynamic Accumulation Process (Ministry of Education of China), China University of Mining and Technology, Xuzhou, Jiangsu 221116 China

## Abstract

The cleat compressibility of coal is a key parameter that is extensively used in modeling the coal reservoir permeability for Coal Bed Methane (CBM) recovery. Cleat compressibility is often determined from the permeability measurement made at different confining pressures but with a constant pore pressure. Hence, this parameter ignores the sorption strain effects on the cleat compressibility. By using the transient pulse decay (TPD) technique, this study presents the results from a laboratory characterization program using coal core drilled from different bedding directions to estimate gas permeability and coal cleat compressibility under different pore pressures while maintaining effective stress constant. Cleat compressibility was determined from permeability and sorption strain measurements that are made at different pore pressures under an effective stress constant. Results show that the cleat compressibility of coal increases slightly with the increase of pore pressure. Moreover, the cleat compressibility of Sample P (representing the face cleats in coal) is larger than that of Sample C (representing the butt cleats in coal). This result suggests that cleat compressibility should not be regarded as constant in the modeling of the CBM recovery. Furthermore, the compressibility of face cleats is considerably sensitive to the sorption-induced swelling/shrinkage and offers significant effects on the coal permeability.

## Introduction

The permeability of coal is one of the most critical parameters for the success of coal bed methane (CBM) recovery from coal reservoirs^1–3^. Coals are viewed as naturally fractured reservoirs with a matrix that is often assumed to have a negligible permeability compared with the fracture system. These fractures in coal are known as cleats. The cleat aperture is sensitive to effective stress. The increase in effective stress tends to decrease the cleat aperture, thereby reducing permeability. As gas desorbs, the coal matrix shrinks and swells with adsorption. The result in shrinkage or swelling is called sorption-induced strain. The reservoir pressure leads to gas desorption and matrix shrinkage, thereby increasing cleat apertures and permeability. However, the counteracting processes of matrix shrinkage and effective stress also operate on cleat apertures during gas production. These situations imply that coal reservoir permeability varies with time.

A number of analytical permeability models have been developed to describe the dynamic permeability behavior during primary (and enhanced) CBM production. The readers are referred to Palmer^1^ and Pan and Connell^4^ for a comprehensive review of these models. Coal permeability is sensitive to reservoir stress conditions and the gas sorption-induced swelling/shrinkage behavior^5–7^. This information has been extensively used in permeability models (e.g., Shiand–Durucan (SD) permeability model):

1 $$k={k}_{0}\,\exp \{-3{C}_{f}(\sigma -{\sigma }_{0})\}$$

2 $$\sigma \,-\,{\sigma }_{0}=-\frac{\nu }{1-\nu }(p-{p}_{0})+\frac{E{\varepsilon }_{V}}{3(1-\nu )},$$

where *k* is the permeability, *k*_0_ is the initial permeability, *C*_*f*_ is the cleat compressibility, *σ* is the effective horizontal stress, *σ*_0_ is the initial effective horizontal stress, *ε*_*V*_ is the volumetric swelling/shrinkage strain, *ν* is the Poisson’s ratio, *E* is the Young’s modulus, *p* is the pore pressure, and *p*_0_ is the initial pore pressure^8,9^.

One important aspect of Eq. (1) is that it is based on a relationship for the change in porosity with pore pressure and stress that is approximate and neglects the influence of sorption strain which will also contribute to the change in porosity with pore pressure. From the definition of the cleat porosity, Connell *et al*.^10^ developed a form of Eq. (1) that included a term due to the sorption strain. This simple extension of Eq. (1) could then explain the observations of coal permeability that reflect sorption strain effects. Connell^11^ presents an extension of the Connell *et al*.^10^ coal permeability model that more generally represents the influences of pore pressure, stress, and sorption strain. The developed model is then tested against a comprehensive suite of laboratory measurements of permeability with respect to pore pressure, stress and with helium, nitrogen, methane and carbon dioxide.

Coal permeability is generally dependent on the mechanical properties of coal. Laboratory experiments were conducted to measure these properties that are required to apply Eqs (1) and (2). Cleat compressibility can be estimated by fitting Eq. (1) to the laboratory measurements on permeability at various pressures or confining pressures. Seidle *et al*.^12^ estimated coal cleat compressibility using Eq. (1) with measurements of permeability to water at different pore pressures while maintaining pressure constant. Pan *et al*.^13^ studied the cleat compressibility of coal from the Sydney Basin in Australia by means of the same method using three different gases, namely, He, CH_4_, and CO_2_. Their results showed that cleat compressibility varies significantly with gas species and pore pressure. However, their results did not show a relationship between cleat compressibility and effective stress. Permeability and cleat compressibility to gas species, pore pressure, effective stress, and temperature were calculated using four gases, namely, He, N_2_, CH_4_, and CO_2_^14^. Connell *et al*.^15^ presented the results of a laboratory characterization using coal core and estimated the properties that are required to apply the SD and Palmer–Mansoori models for coal permeability. Thereafter, these permeability measurements were used to estimate the cleat compressibility by fitting the Seidle model to the observations. Accordingly, cleat compressibility plays an important role in determining the sensitivity of the permeability response to pressure drawdown and matrix shrinkage due to gas desorption. For example, a strong matrix shrinkage behavior may not lead to significant permeability rebound if the cleat compressibility is minimal.

In permeability calculation and reservoir simulation, cleat compressibility is often treated as constant^16–18^. However, Pan *et al*.^1^ estimated cleat compressibility using permeability measurements over a range of pressures and determined that such compressibility was not constant with respect to pressure. Pan *et al*.^1^ compared the calculated permeability using the measured variation by assuming that cleat compressibility was constant. Accordingly, significant differences exist between the two sets of results. Robertson and Christiansen^19^ used the laboratory measurements of permeability to estimate cleat compressibility. These researchers determined that cleat compressibility was not constant. Other studies also revealed that cleat compressibility is not constant and generally varies with pore pressure and effective stress^1,2,20–22^. Cleat compressibility may also change exponentially with respect to the effective stress change for certain types of coal^5,16^.

Laboratory experiments were conducted to measure gas permeability and cleat compressibility with confining pressure at a constant pore pressure^13–15,23^. By maintaining pore pressure constant, the permeability measurements were not complicated by gas sorption-induced swelling effects. However, the underlying mechanism of cleat compressing during the CBM recovery is still unclear. Moreover, the relationship of gas permeability and cleat compressibility with sorption-induced swelling/shrinkage has yet to be substantially understood.

This study uses the transient pulse decay (TPD) technique to present the results of a laboratory characterization program using coal core to measure the gas permeability of coal, as well as estimate coal cleat compressibility under different pore pressures while maintaining effective stress constant. Moreover, the effect of gas sorption-induced swelling/shrinkage on the evolution of coal permeability and cleat compressibility will be discussed symmetrically based on the combined experimental measurements and analytical calculations.

## Experimental principle

**Figure 1:**
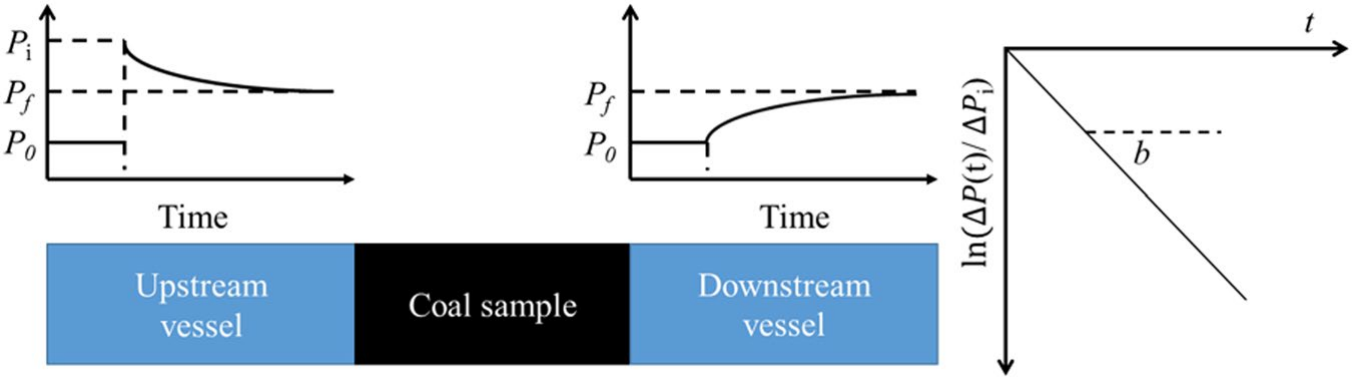
Schematic diagram that describes the principle of the TPD method.

The TPD method was first proposed by Brace *et al*.^24^. This method involves observing the decay of a differential pressure between upstream and downstream cylinders across the sample. This pressure decay is combined with the cylinder volumes in the analysis to relate the flow through the sample, thereby determining the permeability^24^. A schematic diagram that describes the principle of the TPD method is shown in Fig. 1. The pressure decay curve can be modeled as follows:

3 $$\frac{{\rm{\Delta }}P(t)}{{\rm{\Delta }}{P}_{i}}=\exp (-bt).$$

Permeability *k* is linked to the time constant *b* as follows:

4 $$b=\frac{kA}{{\mu }{\boldsymbol{\beta }}L}(\frac{1}{{V}_{u}}+\frac{1}{{V}_{d}}),$$

where Δ*P*(*t*) is the pressure difference between the upstream and downstream cylinders that is measured by a differential pressure transducer; Δ*P*_*i*_ is the pressure difference between the upstream and downstream cylinders at the initial stage, *t* is the time, *k* is the permeability, *A* is the cross-sectional area of the core used, *μ* is the gas viscosity at the test condition (i.e. can be calculated using the NIST web book at http://webbook.nist.gov/chemistry/fluid/), *β* is the compressibility of the fluid in the cylinder, *L* is the length of the core, and *V*_*u*_ and *V*_*d*_ are the volumes of the upstream and downstream cylinders, respectively.

The calculation process includes determining *b* based on the logged differential pressure versus time curve. Thereafter, the permeability *k* can be calculated using Eqs (3) and (4).

## Experimental setup and procedure

**Figure 2:**
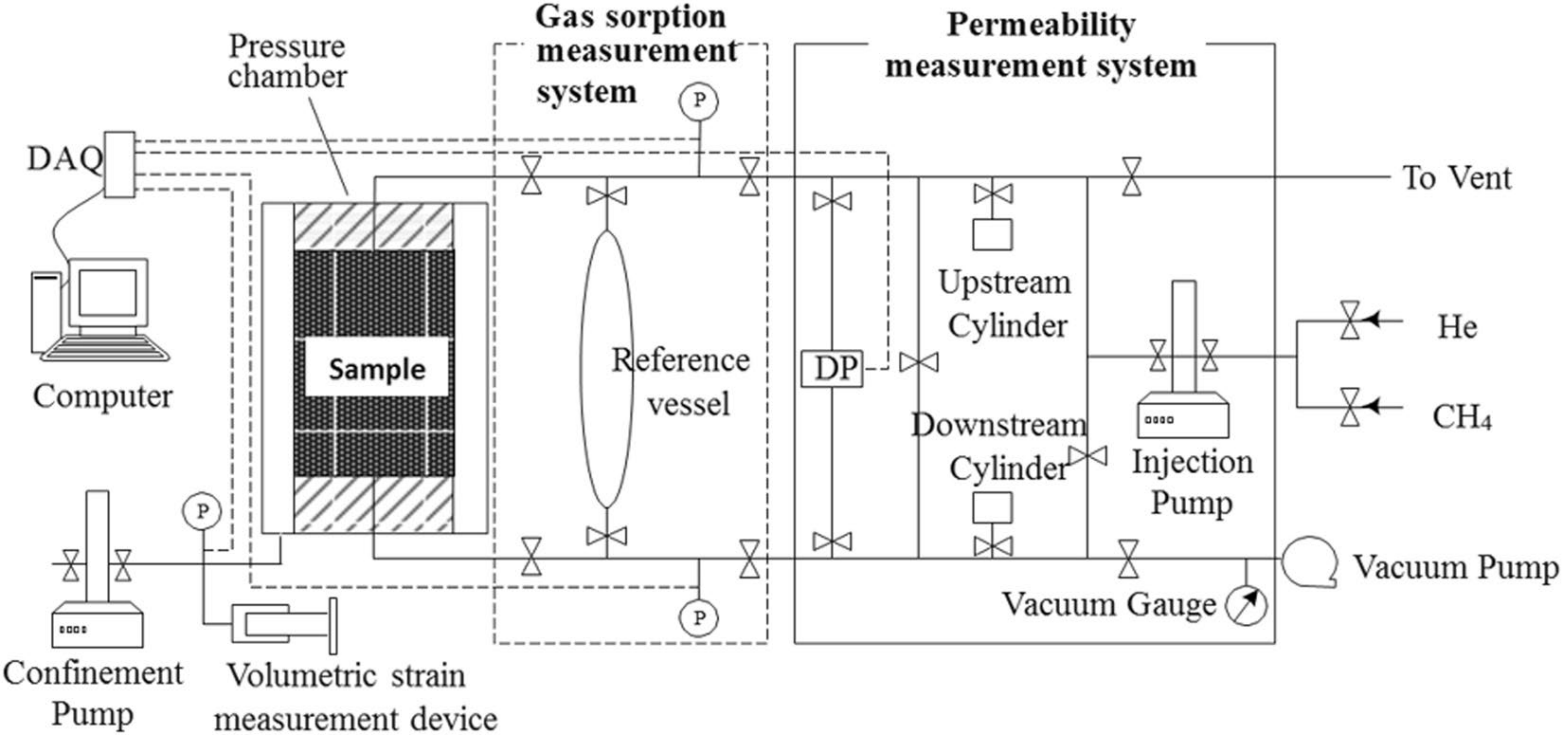
Schematic diagram of the TPD testing device.

Figure 2 shows the schematic diagram of the TPD testing device used for this study. A pressure chamber was used for the experimental measurement of gas permeability under hydrostatic pressure conditions. A confinement pump was used to apply the confining pressure. The core sample, which is nearly 25 mm in diameter and 50 mm in length, is wrapped with a thin lead foil; thereafter, a heat shrinkable tube before it is installed in the pressure chamber. The thin lead foil is used to prevent gas diffusion from the core to the confining fluid at high pressures^25^. The apparatus is engineered to sustain a maximum pore and confining pressures of 16.7 MPa and 25.0 MPa, respectively. The sample chamber and other parts of the apparatus are maintained in the same constant temperature during the experiment. The details of the TPD testing device are provided in other studies^26^.

**Figure 3:**
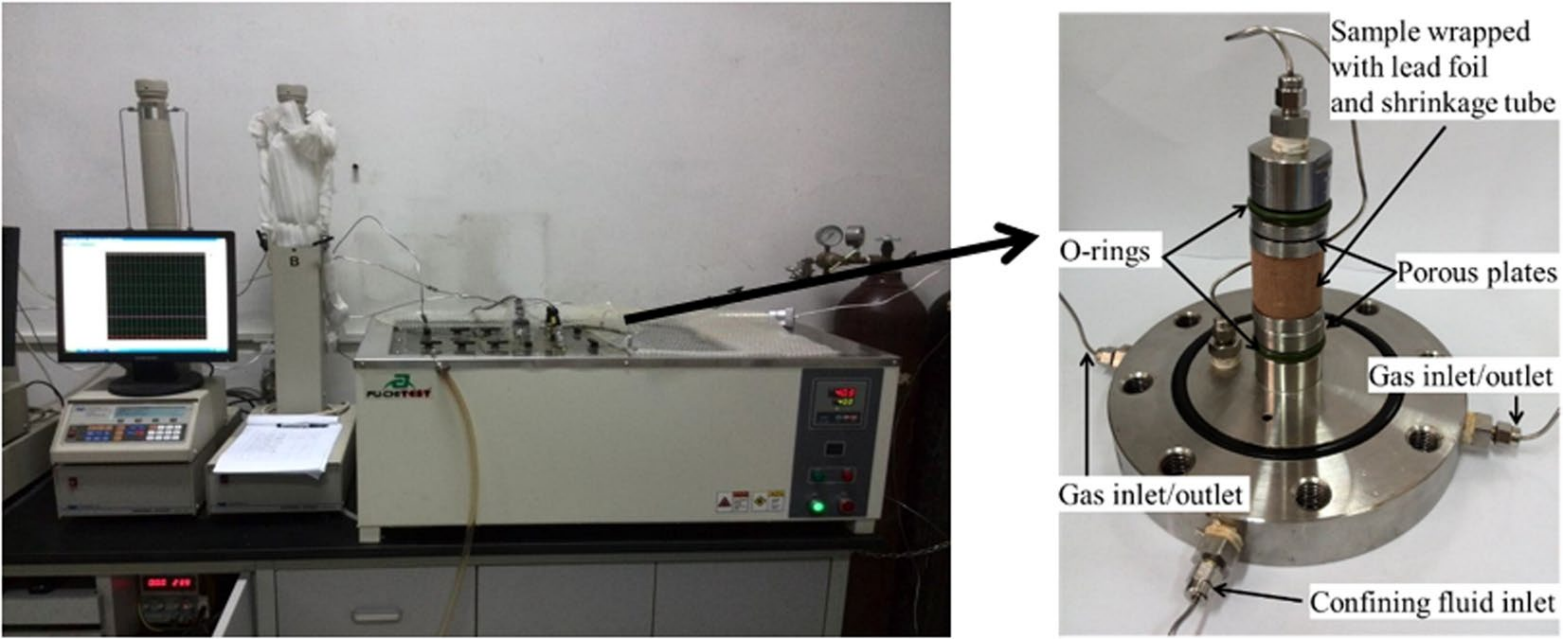
TPD testing device and internal structure of the core holder.
- Confining pressure is applied to a set value using the confinement pump.
- The vacuum pump (for 24 h) is turned on to remove the residual moisture and air in the tube system and sample).
- Gas is injected into the system using a pump at a constant pore pressure (e.g., 3.0 MPa) and the confining pressure (e.g., 8.0 MPa) is adjusted to maintain a constant effective pressure (i.e., 5.0 MPa) before commencing gas injection.
- The valve connected to the tube system and pressure chamber (the void volume has been calibrated first) is opened, thereby enabling the sample to begin adsorbing.
- The gas volume in the injection pump is traced; as the gas volume remains unchanged, the adsorption of the sample reaches equilibrium, thereby enabling the adsorption amount to be calculated.
- In the process of gas adsorption, the volumetric strain measurement device is adjusted to maintain a constant confining pressure.
- When the sample reaches adsorption equilibrium at each pore pressure, the upstream and downstream tubing are separated by closing the valve between the injection pump and downstream cylinder. The pulse pressure is imposed to the core using the injection pump.
- Permeability can be calculated using the TPD method based on the time curve of the pressure difference between the upstream and downstream pumps.
- Changing gas injection pressurecan measure gas adsorption amount of the sample and permeability at different pore pressures.

The testing procedure is presented as follows:

- The prepared core sample (dried or with different water contents) is assembled in the pressure chamber (see Fig. 3).

## Sampling and sample preparation

**Figure 4:**
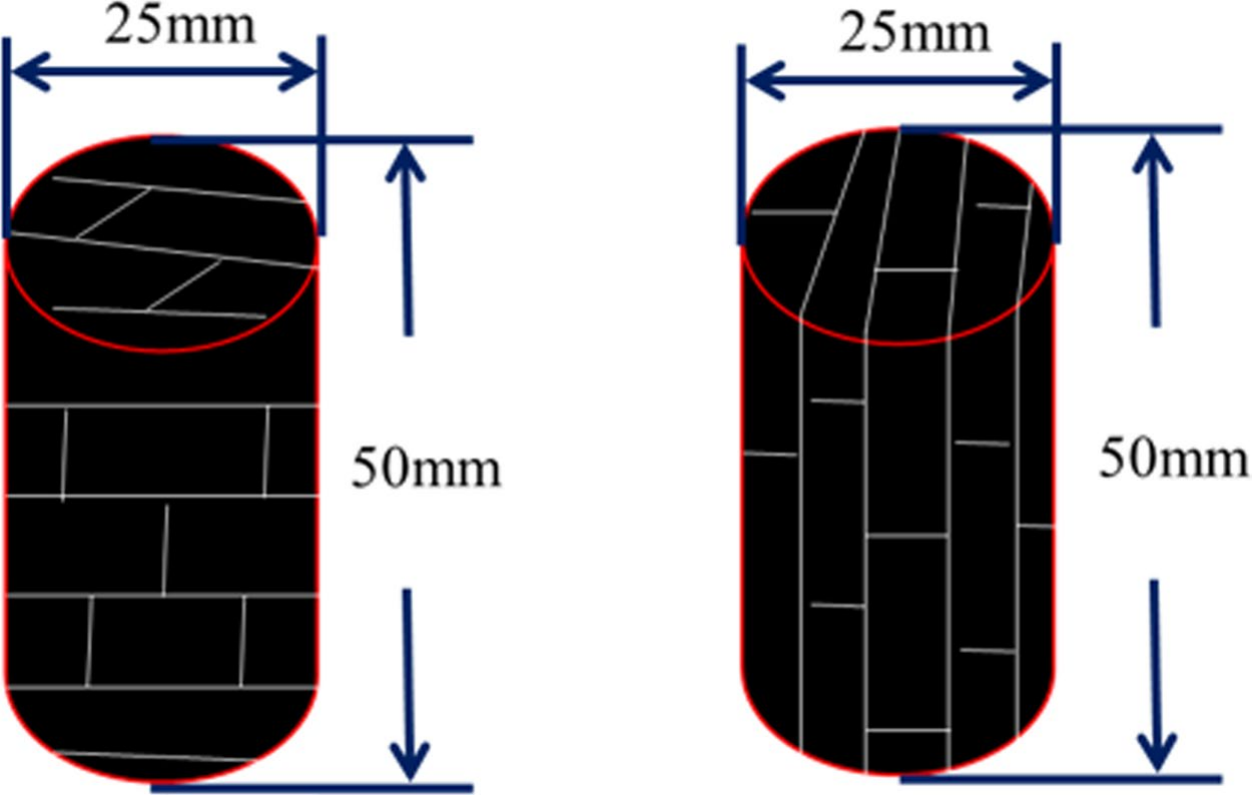
Schematic diagram of the coal core.

The cores of the coal used in this study were obtained from the Chengzhuang coal mine in Qinshui Basin, China. Qinshui Basin is one of the focal areas for CBM exploration and production in China. The adsorption and fluid flow characteristics of coals from this basin have been extensively investigated^27,28^. Table 1 presents a summary of the coal sample’s physical properties. The raw sample was cored to a cylindrical shape with 25 mm in diameter and 50 mm in length in perpendicular (Sample *C*) and parallel (Sample *P*) to the bedding plane in the face cleat direction (see Fig. 4). Samples *C* and *P* generally represent the butt and face cleats, respectively, in coal. Meanwhile, the two core ends were collected, crushed, and ground from 60 to 80 mesh for coal petrology and proximate analyses. CH_4_ and CO_2_ were used for measurements. The purity of the gas was 99.995%. All measurements were conducted at a constant temperature of 40 °C. To eliminate the impact of sorption-induced swelling/shrinkage on permeability, all tests were conducted at a constant effective stress of 5.0 MPa. These tests were controlled by simultaneously tracking the gas injection and confining pressures.

**Table 1:** Summary of the measurements of the coal sample’s physical properties.

Sample name	Bulk density (g/cm^3^)	*Ro*, *max* (%)	Mineral free maceral composition (%)			M_ad_ (%)	V_daf_ (%)	A_d_ (%)	M_e_ (%)
			Exinite	Vitrinite	Inertinite				
*C*	1.36	2.87	6.05	62.57	23.19	1.98	7.33	14.75	4.18
*P*	1.39								

Sample *C* Sample *P*.

## Results and Discussion

### Gas adsorption and sorption strain

**Figure 5:**
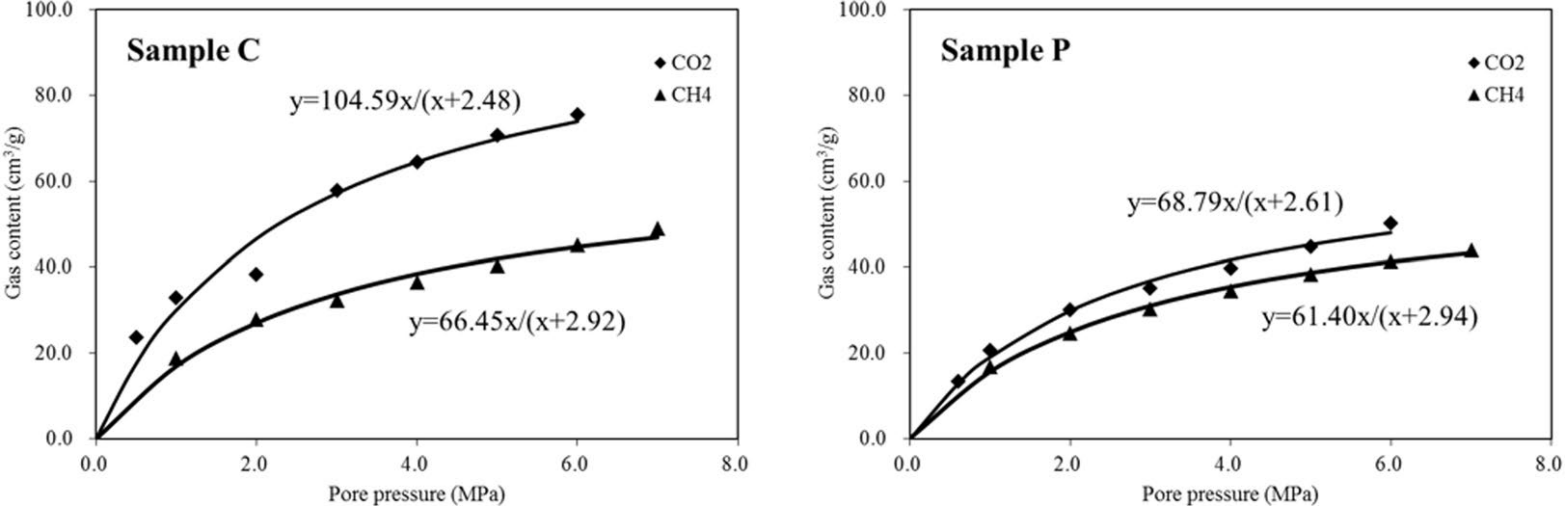
Measurements of the gas content for each adsorbing gas with respect to pore pressure.

Figure 5 shows the measured gas content for each sample with respect to pore pressure after equilibration. The results presented in Fig. 5 show that the gas content increases as the pore pressure increases. Given the same pore pressure, the content of CO_2_ is constantly larger than that of CH_4_, thereby indicating that the adsorption capacity of the former is stronger than the latter.

The Langmuir isotherm was used to model the measured isotherms. This model can be presented as follows:

5 $${Q}_{c}=\frac{{V}_{L}p}{p+{P}_{L}},$$

where *Q*_*c*_ is the gas content, *p* is the pore pressure, *V*_*L*_ and *P*_*L*_ are the Langmuir volume and pressure, respectively.

The measured gas adsorption curves that were obtained by fitting the measurements to the Langmuir model are shown in Fig. 5. Table 2 lists the resultant Langmuir constants for coals. Evidently, the results are different between Samples *C* and P, although such results were obtained from the same coal. The reason for such difference is that Samples *C* and *P* were drilled from the perpendicular direction, thereby possibly leading to differences in internal structures. This result can also be confirmed by the bulk density shown in Table 1. The bulk density of Sample *C* is smaller than that of Sample *P*; hence, the porosity of the former is larger than that of the latter. Therefore, the gas content of Sample *C* is larger than that of Sample *P*. In addition, the difference in the adsorption content of CO_2_ between the two coal samples is larger than that of CH_4_ due to the strong adsorption capacity of the former.

**Table 2:** Summary of the Langmuir constants for the measurements presented in Fig. 5.

Sample	Gas type	*V*_*L*_(cm^3^/g)	*P*_*L*_(MPa)
C	CO_2_	104.59	2.48
	CH_4_	66.45	2.92
P	CO_2_	68.79	2.61
	CH_4_	61.40	2.94

**Figure 6:**
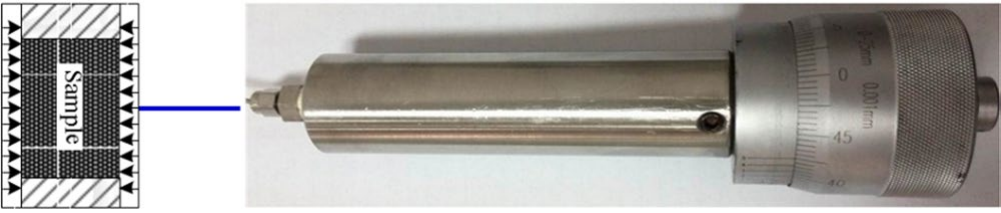
Volumetric strain measurement device.

As the sample swells, the volumetric strain measurement device (see Fig. 6) is adjusted to maintain a constant confining pressure. Thus, the confining fluid volume change that is detected by the device can be used to calculate the swelling of the sample as follows:

6 $${\varepsilon }_{b}=\frac{{\rm{\Delta }}{V}_{f}}{{V}_{c}},$$

where *ε*_*b*_ is the bulk volumetric strain, *ΔV*_*f*_ is the amount of change in the confining fluid volume, and *V*_*c*_ are the core sample volume.

The pore and confining pressures act to compress the samples. Accordingly, these effects should be deducted from the strain measurements to estimate the swelling due to gas adsorption^15^. The volumetric strain can be described by the following relationship based on Jaeger *et al*.^29^:

7 $${\varepsilon }_{bs}={\varepsilon }_{b}+\frac{1}{K}({p}_{C}-\alpha p),$$

where *ε*_*bs*_ is defined as the sorption strain, *ε*_*b*_ is the bulk volumetric strain, *K* is the bulk modulus, *p*_*c*_ is the confining pressure, and *a* is the Biot coefficient.

The confining pressure remained constant by adjusting the volumetric strain measurement device during the gas adsorption process. The bulk modulus can be calculated from the Young’s modulus and Poisson’s ratio. The Biot coefficient is equal to one. All tests were conducted at a constant effective stress of 5.0 MPa.

**Figure 7:**
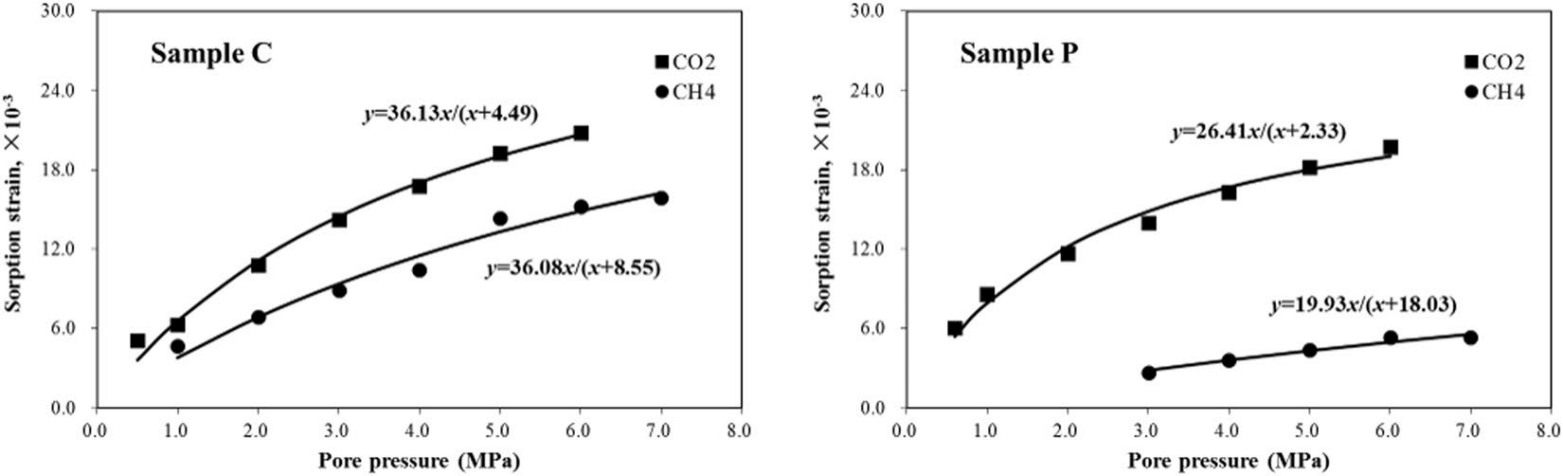
Sorption strain with respect to the pore pressure after adsorption has equilibrated.

Figure 7 presents the sorption strains calculated using Eq. (7) with respect to pore pressure after equilibration. A Langmuir-type model was used to describe these measurements. The least squares fits of this model with the measurements are presented in Fig. 7. The sorption strain with respect to the pore pressure model can be presented as follows:

8 $${\varepsilon }_{bs}=\frac{{\varepsilon }_{\max }p}{p+{P}_{\varepsilon }},$$

where *ε*_*max*_ is defined as the Langmuir sorption strain and *P*_*ε*_ is the Langmuir pressure when the maximal Langmuir sorption strain amount is 50%. Table 3 presents the results.

**Table 3:** Summary of the Langmuir sorption strain and pressure for the measurements presented in Fig. 7.

Sample	Gas type	*ε*_*max*_ (×10^−3^)	*P*_*ε*_ (MPa)
C	CO_2_	36.13	4.49
	CH_4_	36.08	8.55
P	CO_2_	26.41	2.33
	CH_4_	19.93	18.03

### Effects of gas sorption-induced swelling/shrinkage on coal permeability

This study measured the permeability of coal by following the gas adsorption measurement after reaching adsorption equilibrium. To measure the permeability, the upstream cylinder is charged to the pressure of approximately 30 kPa above the pore pressure in the core sample. By contrast, the downstream cylinder pressure is charged to approximately 30 kPa below thepore pressure. Both cases used the same gas that was pre-adsorbed in the core sample. Thereafter, the valves between the upstream and downstream cylinders and core sample are opened to enable the gas to flow through the core sample from the two cylinders. This study used four pore pressure steps (i.e., 1.0, 2.0, 3.0, and 4.0 MPa) to obtain the permeability at a constant effective stress. Permeability was calculated using Eqs (3) and (4) based on the logged differential pressure versus time curve.

**Figure 8:**
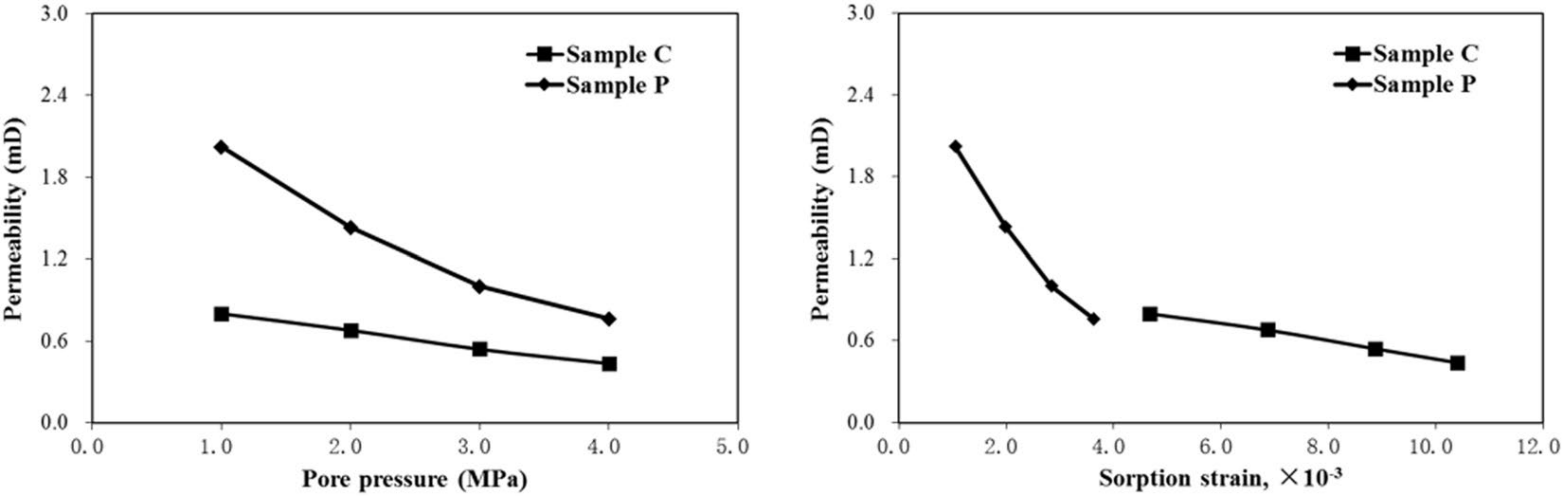
Results of the permeability measured using CH_4_ with respect to pore pressure and sorption strain.

The results of the permeability measurement using CH_4_ with respect to pore pressure and sorption strain at a constant effective stress of 5.0 MPa are shown in Fig. 8. For the pore pressure range of 1.0–4.0 MPa, the permeability measured by Sample P is higher than that measured using Sample C at the same pore pressure. This result indicates that the face cleats substantially contribute to coal permeability. Moreover, the permeabilities measured using Samples P and C decreased with increasing pore pressure and sorption strain, although the permeability of the former decreases more rapidly than that of the latter. The permeability decrease is mainly attributed to the decrease in coal cleat width due to gas adsorption-induced coal matrix swelling. Moreover, the effective stress has no effect in this particular case because of the constant effective stress condition.

In general, increasing the pore pressure will increase the stress acting on the cleats, thereby resulting in cleat compressibility that increases cleat width. Furthermore, increasing the pore pressure will cause gas adsorption, thereby possibly decreasing the cleat width. The former is a mechanical effect that tends to increase permeability, whereas the latter reflects the sorption-induced strain impact that tends to decrease permeability. However, all experiments in this research were conducted under a constant effective stress condition. When the pore pressure increases, the confining pressure also increases; hence, their difference is 0 to maintain a constant effective stress. That is, the mechanical effect can be eliminated by considering the impact of the sorption-induced strain. Therefore, the permeabilities measured using Samples P and C decrease with the increase in sorption strain at a constant effective stress (i.e., 5.0 MPa) due to the decrease in cleat width.

### Effect of gas sorption-induced swelling/shrinkage on the compressibility of coal cleat

In previous studies, cleat compressibility is determined from the permeability measurements made at different confining pressures but with constant pore pressure^13–15^. The objective of holding the pore pressure constant was to avoid changes in gas content and effects of sorption strain on the estimated cleat compressibilities^15^. Therefore, the effect of sorption-induced swelling/shrinkage on cleat compressibility was disregarded. In the current study, cleat compressibility will be determined from the permeability measurements made at different pore pressures but with constant effective stress. Thus, the effect of coal sorption-induced swelling/shrinkage on the evolution of permeability and cleat compressibility can be analyzed.

The following equation is obtained by substituting Eqs (2) (8) into Eq. (1):

9 $$k={k}_{0}\exp \{{C}_{f}[\frac{3\nu }{1-\nu }(p-{p}_{0})-\frac{E}{(1-\nu )}(\frac{{\varepsilon }_{\max }p}{p+{P}_{\varepsilon }}-\frac{{\varepsilon }_{\max }{p}_{0}}{{p}_{0}+{P}_{\varepsilon }})]\}.$$

Numerous coal permeability models have been developed over the past few decades. The models can be divided into uniaxial strain conditions, constant volume conditions and triaxial stress using different boundary conditions. The P&M and S&D models were developed based on uniaxial conditions. Therefore, Eq. (9) applies only to uniaxial strain with constant vertical external stress conditions.

Lu *et al*.^30^ developed a new permeability model that considered the effective stress and matrix sorption (desorption) deformation under triaxial conditions. They developed the permeability model using gas sorption (desorption)-induced deformation, effective stress and porosity:

10 $$\frac{k}{{k}_{0}}=\exp \{-3{C}_{f}[(\bar{\sigma }-{\bar{\sigma }}_{0})-(p-{p}_{0})+f\frac{E}{3(1-2\nu )}\frac{{\varepsilon }_{\max }{P}_{\varepsilon }(p-{p}_{0})}{(p+{P}_{\varepsilon })({p}_{0}+{P}_{\varepsilon })}]\}$$

Where $\bar{\sigma }$ is the mean stress, ${\bar{\sigma }}_{0}$ is the initial mean stress, and *f* is the internal swelling (shrinking) partition, which ranges from 0 to 1. The value for *f* is determined by and may be controlled by the coal structure^10^. When the effective stress is constant, the permeability model can be expressed as follows:

11 $$\frac{k}{{k}_{0}}=\exp \{-3{C}_{f}[f\frac{E}{3(1-2\nu )}\frac{{\varepsilon }_{\max }{P}_{\varepsilon }(p-{p}_{0})}{(p+{P}_{\varepsilon })({p}_{0}+{P}_{\varepsilon })}]\}$$

Eq. (11) can be further re-written as follows:

12 $${C}_{f}=\,\mathrm{ln}\,\frac{k}{{k}_{0}}/\{-3[f\frac{E}{3(1-2\nu )}\frac{{\varepsilon }_{\max }{P}_{\varepsilon }(p-{p}_{0})}{(p+{P}_{\varepsilon })({p}_{0}+{P}_{\varepsilon })}]\}.$$

Thus, the cleat compressibility *C*_*f*_ can be obtained by fitting the experimental data using Eq. (12).

**Figure 9:**
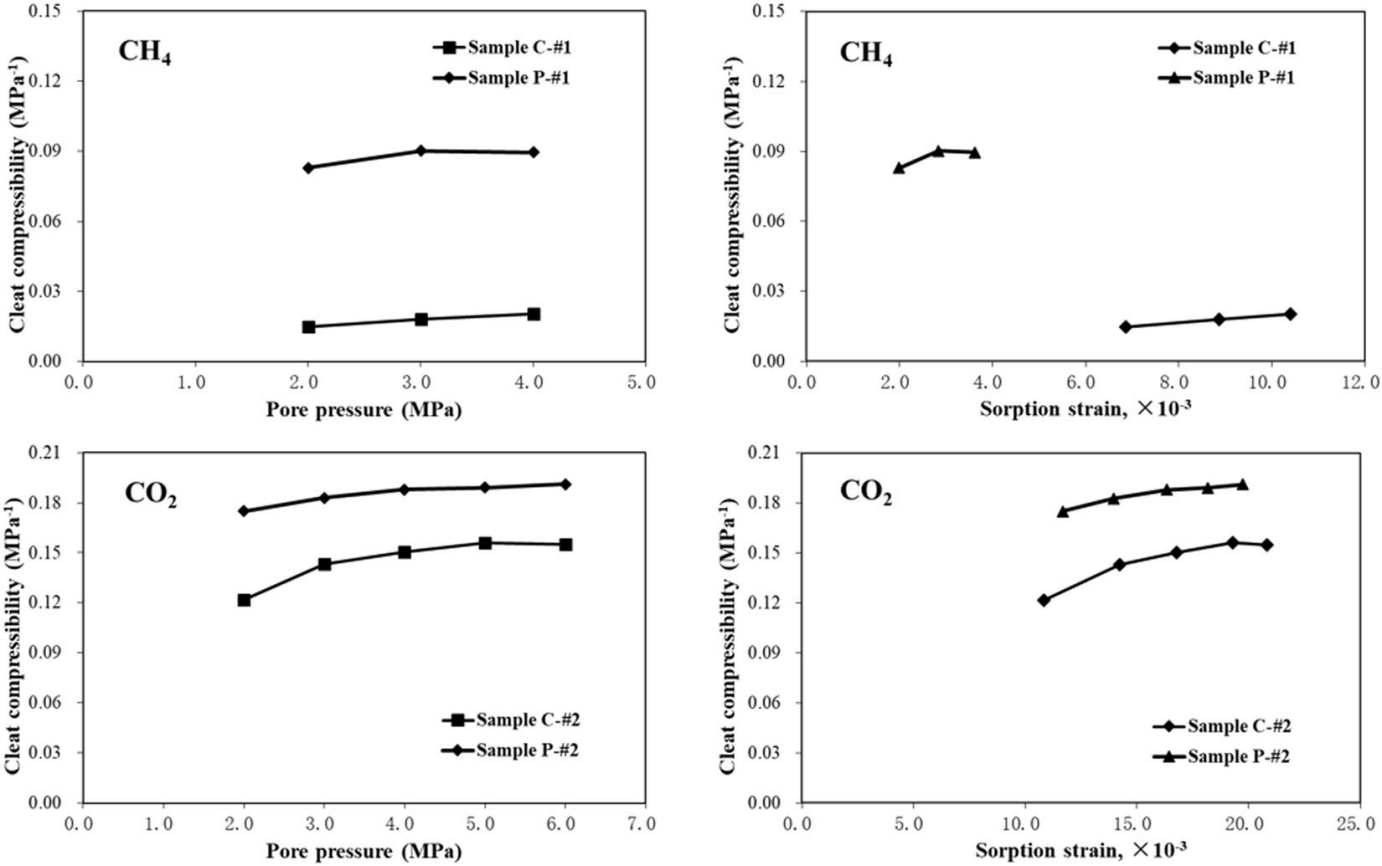
Cleat compressibility of Samples *C* and *P* with respect to pore pressure and sorption strain.

The cleat compressibility that was calculated at various pore pressures for Samples *C* and *P* are plotted in Fig. 9. Evidently, cleat compressibility increases slightly with an increase in pore pressure and sorption strain. The adsorption of gas causes matrix swelling, thereby slightly decreasing the cleat width (or porosity) at given experimental conditions. Seidle *et al*.^12^ reported that cleat compressibility increases with a decrease in porosity if porosity change with respect to stress change is constant. However, cleat compressibility tends to increase smoothly when the pore pressure increases. Thus, the impact of sorption-induced swelling/shrinkage on permeability increases with increasing pore pressure and sorption strain. However, this effect is relatively stronger at the low pore pressure stage than at the high pore pressure stage. This means that at low pore pressure stage, the impact of effective stress on permeability increases more quickly with pore pressure increase. While at high pore pressure stage, the cleat compressibility increases more slowly with increase of pore pressure. The reason for these behaviours may be because that the resistance of coal matrix with increasing pore pressure decreases gradually at low pressure stage, while it increases again at high pressure stage.

It can be seen from Fig. 9 that the cleat compressibility of Sample *P* is larger than that of Sample *C*, that is because the internal pore structures of the coal samples are different from one to another (see Fig. 4). The effect of the sorption-induced swelling/shrinkage on permeability is more pronounced in parallel to the bedding plane in the face cleat direction than in the perpendicular one.

It also can be seen from Fig. 9 that cleat compressibility using different gases differ quite significantly. Cleat compressibility measured using CO_2_ is larger than that measured using CH_4_ at the same pore pressure. One possible explanation for this gas species dependence is that it is related to adsorption induced coal swelling. The adsorption of gas causes the matrix swelling, part of which decreases the cleat porosity at the experimental conditions. As we know, the adsorption of carbon dioxide is stronger than that of methane, resulting in greater adsorption strain.

A range of properties has to be estimated when the SD and Palmer-Mansoori models are applied to evaluate coal reservoir permeability. Among these parameters, cleat compressibility is a key property that is extremely difficult, if not impossible, to measure directly. This parameter plays an important role in determining the sensitivity of the coal permeability response to pressure drawdown and matrix shrinkage due to gas desorption during the CBM recovery. In the SD model, cleat compressibility is often assumed to be constant although pore pressure may be changing. However, the results indicated in Fig. 9 reveals that cleat compressibility should not be regarded as a constant. This observation is consistent with other studies. Pan *et al*.^13^ suggested variable cleat compressibility may have significant impact on permeability predictions. The results obtained by Zheng *et al*.^14^ show that cleat compressibility is also strongly dependent on pore pressure. McKee *et al*.^16^ proposed a relationship for the cleat compressibility with respect to the effective horizontal stress.

Permeability is a key property for gas production however measurements on core samples are rarely representative of field conditions due to a range of issues. The objective of measuring permeability in this characterisation program is to estimate the cleat compressibility needed to apply Eq. (1) in reservoir simulation studies. However, there could be differences between the values determined from the laboratory testing under hydrostatic conditions and what would apply in the reservoir where uniaxial strain, constant vertical stress conditions apply. Note that the cleat compressibility discussed in this study merely illustrates the impact from sorption-induced swelling/shrinkage. When cleat compressibility is used to calculate coal permeability, the impact from gas species, effective stress, pore pressure, and temperature should be considered. This consideration is worthy of further investigation.

## Conclusions

This study presents an alternative method to investigate the effects of sorption-induced swelling/shrinkage on coal permeability and cleat compressibility. The TPD technique was employed for the laboratory characterization of the coal core to measure coal permeability with gas adsorption under a constant effective pressure that can be maintained by adjusting the confining pressure. The constant effective pressure can minimize stress-induced deformation, thereby providing insight into experiments that investigate the effects of adsorption-induced coal swelling/shrinkage on coal permeability and cleat compressibility.

A series of measurements of gas permeability under a constant effective pressure condition with different pore pressures have been conducted using the coal core samples from the Qinshui Basin. The sorption strain measured with respect to pore pressure is well described using the Langmuir-type model. Meanwhile, permeability was determined by means of the logged differential pressure versus time curve. Under a constant effective stress condition, the permeabilities measured using Sample P (featured by the face cleats) and Sample C (featured by the butt cleats) decreased with increasing pore pressures. The decrease in permeability is mainly attributed to a decrease in coal cleat width due to gas adsorption-induced coal matrix swelling. Furthermore, the permeability measured using Sample P is higher than that measured using Sample C under the same pore pressure. This result implies that the face cleats contribute substantially to coal permeability.

Cleat compressibility was determined from the measurements of permeability and sorption strain made at different pore pressures under a constant effective stress. The results show that cleat compressibility increases slightly with an increase of pore pressure, whereas it tends to increase smoothly when the pore pressure increases. The results suggest that cleat compressibility should not be regarded as constant. In addition, the effect of sorption-induced swelling/shrinkage on cleat compressibility is more pronounced in Sample P compared with Sample C. This result indicates that the compressibility of face cleats may be considerably sensitive to sorption-induced swelling/shrinkage.
